# Supplementary material for: Distinct gut microbiome shifts in the NICU influence later atopic dermatitis development
Source: medRxiv. 2025 Sep 4:2025.09.03.25334680. Preprint. [Version 1] doi: 10.1101/2025.09.03.25334680 (PMC12443057; doi:10.1101/2025.09.03.25334680)
Supplement: Supplement 2 [file media-2.docx]

**Supplemental Methods**

*Subjects and Sample Collection*

Neonates who spent time in the Inova Fairfax Neonatal Intensive Care Unit (NICU) were enrolled in an observational longitudinal microbiome cohort study, as previously described^1,2^. The study was Institutional Review Board approved (WCG IRB 1300205) and parental informed consent was obtained. Neonates were enrolled within the first week of life and had an anticipated stay in the NICU of >5 days. While in the NICU stool samples were collected for microbiome analysis up to twice a week, and stored as whole stool at -80°C until analysis. Detailed demographic and clinical data was collected including delivery mode, gestational age, maternal peripartum antibiotic use and infant antibiotics.

After discharge from the NICU, follow-up surveys reporting health, illnesses, diet and a variety of exposures were collected approximately every 3–6 months until approximately 3 years of age (corrected for gestational age), accompanied by a stool sample collected by previously validated methods^3^. One final follow up survey was sent to every family when the child was around age 5 years of age, with more specifics regarding atopic diagnoses. For this, a slightly modified ISAAC questionnaire was used to determine the diagnosis of atopic dermatitis (AD), allergic rhinitis, asthma, or food allergies^4^.

For this current study, neonates from the larger cohort were included if they had at least 1 stool sample from before 1 month of life, at least 2 stool samples overall and completed the final survey regarding allergic disease.

*DNA Extraction*

DNA was extracted from fecal samples in two stages. First, approximately 50 mg of fecal material and 650 μL MBL lysis buffer from the PowerMicrobiome DNA/RNA EP Kit (Qiagen) were added to Lysis Matrix E (LME) tubes (MP Biomedicals). LME tubes were transferred to a Precelleys 24 Tissue Homogenizer (Bertin Technologies) and fecal samples were homogenized, centrifuged, with the resultant supernatant transferred to a deep-well 96-well plate. The second stage consisted of DNA isolation from the above supernatant using the MagAttract PowerMicrobiome DNA/RNA EP Kit (Qiagen) on an automated liquid handling system as detailed by the manufacturer (Eppendorf).

*Shotgun Metagenomic Sequencing*

Total gene content of the microbiome was assessed through shotgun metagenomic sequencing. Metagenomic libraries were constructed from 100 ng of DNA as starting material using the Illumina DNA Prep kit. Illumina DNA/RNA UD Indexes were used to add sample-specific sequencing indices to both ends of the libraries. An Agilent 4200 TapeStation system with High Sensitivity D5000 ScreenTape (Agilent Technologies, Inc) was used to verify quality and assess final library size. A positive control (MSA-2002 20 Strain Even Mix Whole Cell Material (ATCC)) and a buffer extraction negative control were included. Metagenomic libraries were normalized and pooled at an equimolar concentration. Final pools were sequenced on a NovaSeq X sequencer using a paired-end (150x150) NovaSeq 25B flow cell across ten lanes (Illumina, Inc).

*Sequence Processing*

*Quality Assessment*. Paired-end sequences were assessed for quality with FastQC and MultiQC^5,6^. Reads then underwent the Whole-Genome Sequence Assembly 2 (WGSA2) protocol from the Nephele platform^7,8^. In brief, reads were processed with fastp and minimal trimming and filtering by ensuring an average read quality of 10, a trim of the 3’ end of the read at a quality of 15 and trimming the 5’ end at a Q score of 20 with additional filtering of reads if they were less than 60bp after trimming, and automatic trimming of adapters^9^. Human reads were decontaminated from sequence data using Kraken2 with a database containing the human and mouse genome^10^.

*Assembly and Gene Annotation*.

Within the WGSA2 pipeline, reads were assembled into contiguous sequences, or contigs, using metaSPAdes.^11^ Reads were recruited back to contigs using bowtie2 and SAMtools to produce information on scaffold coverage and quality^12,13^. Protein coding regions (CDS) were predicted from assembled scaffolds using Prodigal^14^. Predicted CDS regions were processed by EggNOG-mapper2 to identify and annotate genes with KEGG Orthology (KO) identifiers^15,16^. Annotated genes were agglomerated into pathways using MinPath. Non-microbial pathways were filtered by identifying all pathways present across all bacteria, fungi and archaea in the KEGG database. Abundances were calculated using VERSE to obtain Transcripts per Million (TPM) at the CDS level and summed to obtain TPM by pathway^17,18^.

*Taxonomic Classification*.

Processed reads were classified taxonomically using Ganon^19^. Three custom, independent databases were built on December 19, 2024 including (1) archaea, bacteria and fungi reference genomes from RefSeq, (2) Viral Complete Genomes from RefSeq and (3) the top 1 species of archaea and bacteria from the Genome Taxonomy DataBase (GTDB). Databases were used in a hierarchical manner, in which both RefSeq databases were used as one database, and if a read could not be classified by RefSeq, it would undergo classification by GTDB. A relative cut-off of 0.2 was used for all samples. After classification by RefSeq and GTDB, the MultiTax package, included with the Ganon package, standardized the calls to the NCBI lineage.

*Microbiome Analysis*

Taxonomy. Ganon classification resulted in 59,191 species across all samples, with samples containing between 29M and 39M (IQR) reads per sample. Species were filtered if they had fewer than 10,000 reads, resulting in 5,895 species, but only removing 0.21% of the total reads in the dataset (remaining Q1: 29M and Q3: 38M). Within samples, a minimum of 0.02% and a maximum of 4.73% of reads were removed (Q1: 0.12%; Q3: 0.25%). Samples underwent rarefaction curves and found that most samples approached an asymptote and thus abundances were normalized to reads per million to maintain all samples. Alpha diversity statistics and beta diversity distance matrices were calculated by the phyloseq package^20^.

Latent Classes. Infants were separated into distinct trajectories of microbiota development, as described by Bray Curtis PCoA 2, utilizing latent class mixed models with the lcmm package in R^21^. The 10 most dominant genera, as well as PCoA 1 and PCoA 2 from Bray Curtis were tested for the presence of divergent trajectories, though only PCoA 2 provided well-distributed, distinct trajectories that also related to AD development. In brief, a model with no separation was built to obtain initial values. Between 2 and 5 separations were performed and fit was compared to the initial values using the Bayesian Information Criterion (BIC) and log-likelihood.

Mediation Analysis. Mediation effects between all variables collected from participants at the subject level (“treatment”) and PCoA Trajectory (“Mediator”) on Atopic Dermatitis (“outcome”) were identified using the mediation package in R^22^. Variables were not considered at all if they had a prevalence less than 10%, or were missing in at least 60% of participants. Several estimates were obtained, including (1) the effect of exposure on the mediator, (2) the effect of exposure on outcome, adjusted for the mediator, (3) effect of treatment on the outcome, and (4) effect of mediator on the outcome. Each effect was estimated with generalized linear models, with additional adjustment for sex. Models 1 and 2 were used in the mediate function to calculate Average Causal Mediation Effects (ACME) and ACME p-values.

Statistical Analysis. Because the microbiota changed significantly over time due to the well-described nature of microbiome development, linear models could not adequately capture the change in features of interest over time. Thus, Generalized Additive Mixed Models from the mgcv package were used for statistical tests occurring over time in order to capture the variation specific to our variables of interest^23^. The model was constructed as follows:
Abundance ~ Variable + s(Days of Life, by= Variable) + s(SubjectID, bs=”re”)

With smoothing of days of life stratified by our variable of interest, and an additional smooth term for subject with a random effect basis. P-values less than 0.01 were considered significant, and significant intervals were calculated with the *marginal effects* package^24^; intervals were corrected for multiple comparisons and an FDR p-value < 0.05 considered significant. Permutational Analysis of Variance was calculated with the adonis2 function in vegan using an interaction term between the variable of interest and Days of Life^25^. Permutations were constrained within subjects using a “series” permutation design.

**References**

1. Chaudhary PP, Myles IA, Zeldin J, et al. Shotgun metagenomic sequencing on skin microbiome indicates dysbiosis exists prior to the onset of atopic dermatitis. *Allergy*. Oct 2023;78(10):2724-2731. doi:10.1111/all.15806

2. Hourigan SK, Subramanian P, Hasan NA, et al. Comparison of Infant Gut and Skin Microbiota, Resistome and Virulome Between Neonatal Intensive Care Unit (NICU) Environments. *Front Microbiol*. 2018;9:1361. doi:10.3389/fmicb.2018.01361

3. Wong WSW, Clemency N, Klein E, et al. Collection of non-meconium stool on fecal occult blood cards is an effective method for fecal microbiota studies in infants. *Microbiome*. Sep 5 2017;5(1):114. doi:10.1186/s40168-017-0333-z

4. Asher MI, Keil U, Anderson HR, et al. International Study of Asthma and Allergies in Childhood (ISAAC): rationale and methods. *Eur Respir J*. Mar 1995;8(3):483-91. doi:10.1183/09031936.95.08030483

5. FastQC: A Quality Control Tool for High Throughput Sequence Data. FastQC: A Quality Control Tool for High Throughput Sequence Data.

6. Ewels P, Magnusson M, Lundin S, Käller M. MultiQC: summarize analysis results for multiple tools and samples in a single report. *Bioinformatics*. Oct 1 2016;32(19):3047-8. doi:10.1093/bioinformatics/btw354

7. Angelova A DD, Subramanian P, Quiñones M, Dolan M, Hurt DE. WGSA2 workflow - a tutorial. *protocolsio*. 2023;doi:doi:10.17504/protocols.io.n92ldm98xl5b/v1

8. Weber N, Liou D, Dommer J, et al. Nephele: a cloud platform for simplified, standardized and reproducible microbiome data analysis. *Bioinformatics*. Apr 15 2018;34(8):1411-1413. doi:10.1093/bioinformatics/btx617

9. Chen S, Zhou Y, Chen Y, Gu J. fastp: an ultra-fast all-in-one FASTQ preprocessor. *Bioinformatics*. Sep 1 2018;34(17):i884-i890. doi:10.1093/bioinformatics/bty560

10. Wood DE, Lu J, Langmead B. Improved metagenomic analysis with Kraken 2. *Genome Biol*. Nov 28 2019;20(1):257. doi:10.1186/s13059-019-1891-0

11. Nurk S, Meleshko D, Korobeynikov A, Pevzner PA. metaSPAdes: a new versatile metagenomic assembler. *Genome Res*. May 2017;27(5):824-834. doi:10.1101/gr.213959.116

12. Bankevich A, Nurk S, Antipov D, et al. SPAdes: a new genome assembly algorithm and its applications to single-cell sequencing. *J Comput Biol*. May 2012;19(5):455-77. doi:10.1089/cmb.2012.0021

13. Li H, Handsaker B, Wysoker A, et al. The Sequence Alignment/Map format and SAMtools. *Bioinformatics*. Aug 15 2009;25(16):2078-9. doi:10.1093/bioinformatics/btp352

14. Hyatt D, Chen GL, Locascio PF, Land ML, Larimer FW, Hauser LJ. Prodigal: prokaryotic gene recognition and translation initiation site identification. *BMC Bioinformatics*. Mar 8 2010;11:119. doi:10.1186/1471-2105-11-119

15. Cantalapiedra CP, Hernández-Plaza A, Letunic I, Bork P, Huerta-Cepas J. eggNOG-mapper v2: Functional Annotation, Orthology Assignments, and Domain Prediction at the Metagenomic Scale. *Mol Biol Evol*. Dec 9 2021;38(12):5825-5829. doi:10.1093/molbev/msab293

16. Kanehisa M, Sato Y, Kawashima M, Furumichi M, Tanabe M. KEGG as a reference resource for gene and protein annotation. *Nucleic Acids Res*. Jan 4 2016;44(D1):D457-62. doi:10.1093/nar/gkv1070

17. Ye Y, Doak TG. A parsimony approach to biological pathway reconstruction/inference for genomes and metagenomes. *PLoS Comput Biol*. Aug 2009;5(8):e1000465. doi:10.1371/journal.pcbi.1000465

18. Zhu Q, Fisher S, Shallcross J, Kim J. VERSE: a versatile and efficient RNA-Seq read counting tool. bioRxiv; 2016.

19. Piro VC, Dadi TH, Seiler E, Reinert K, Renard BY. ganon: precise metagenomics classification against large and up-to-date sets of reference sequences. *Bioinformatics*. Jul 1 2020;36(Suppl_1):i12-i20. doi:10.1093/bioinformatics/btaa458

20. McMurdie PJ, Holmes S. phyloseq: an R package for reproducible interactive analysis and graphics of microbiome census data. *PLoS One*. 2013;8(4):e61217. doi:10.1371/journal.pone.0061217

21. Proust-Lima C, Philipps V, Liquet B. Estimation of Extended Mixed Models Using Latent Classes and Latent Processes: The R Package lcmm. *Journal of Statistical Software*. 06/01 2017;78(2):1 - 56. doi:10.18637/jss.v078.i02

22. Tingley D, Yamamoto T, Hirose K, Keele L, Imai K. mediation: R Package for Causal Mediation Analysis. *Journal of Statistical Software*. 09/02 2014;59(5):1 - 38. doi:10.18637/jss.v059.i05

23. Wood SN. *Generalized Additive Models: An Introduction with R, Second Edition (2nd ed.). Chapman and Hall/CRC.* . 2017.

24. Arel-Bundock V, Greifer N, Heiss A. How to Interpret Statistical Models Using marginaleffects for R and Python. *Journal of Statistical Software*. 11/30 2024;111(9):1 - 32. doi:10.18637/jss.v111.i09

25. Oksanen J BF, Kindt R, Legendre P, Minchin P, O'Hara B, Simpson G, Solymos P, Stevens H, Wagner H. Vegan: Community Ecology Package. 2022;
